# Metabolic syndrome increases the risk for premature employment exit: A longitudinal study among 60 427 middle-aged and older workers from the Lifelines Cohort Study and Biobank

**DOI:** 10.5271/sjweh.4113

**Published:** 2023-11-01

**Authors:** Katharina Runge, Sander KR van Zon, Kène Henkens, Ute Bültmann

**Affiliations:** 1 Netherlands Interdisciplinary Demographic Institute, The Hague, The Netherlands.; 2 Department of Health Sciences, Community and Occupational Medicine, University of Groningen, University Medical Center Groningen, Groningen, The Netherlands.; 3 Faculty of Social and Behavioral Sciences, University of Amsterdam, Amsterdam, The Netherlands.

**Keywords:** cardio-metabolic risk factor, early retirement, unemployment, work disability

## Abstract

**Objectives:**

This study aimed to examine whether (i) metabolic syndrome (MetS) increases the risk for premature employment exit and (ii) a dose–response relationship exists between an increasing number of MetS components and premature employment exit among middle-aged and older workers.

**Methods:**

A sample of N=60 427 Dutch workers (40–64 years old) from the Lifelines Cohort Study and Biobank were examined using data from five measurement waves during a total median follow-up time of 4.2 years. MetS components were based on physical measures, blood markers, and medication use. Premature employment exit types (ie, unemployment, work disability, and early retirement) were determined using questionnaires. MetS and number of MetS components were examined as risk factors for premature employment exit using competing risk regression analysis.

**Results:**

MetS significantly increased the risk for work disability [adjusted sub distribution hazard ratio (SHR) 1.78, 95% confidence interval (CI) 1.54–2.05] and unemployment (adjusted SHR 1.16, 95% CI 1.06–1.26). A clear dose–response relationship was found for an increasing number of MetS components and work disability. No associations were found between MetS (components) and early retirement after adjusting for sociodemographic factors.

**Conclusions:**

MetS was identified as a modifiable early-stage cardio-metabolic risk factor especially for work disability and, to a lesser extent, for unemployment. Further, a clear dose–response relationship was found between an increasing number of MetS components and work disability. MetS interventions and prevention might help to prolong working lives. More awareness is needed among employers and occupational health professionals about the premature employment exit risk faced by middle-aged and older workers with MetS.

Many Western countries currently face a combination of increasing life expectancy, decreasing fertility rates, and at the same time a large number of older workers from the baby boomer generation transitioning into retirement (1). At the societal level, it is important to maintain older workers in employment longer to counteract labor market shortages. This need is reflected in extending working life policies aimed at increasing statutory retirement ages, making early retirement less attractive, and promoting working beyond pension age (2). At the individual level, it is critical to ensure that healthy and meaningful working years are added to the working career (3). To date, however, many workers still exit employment before reaching the state pension age, eg, through unemployment, work disability, or early retirement (4). Premature employment exit, especially when involuntary, is associated with adverse mental and physical health outcomes (5–8). For longer working lives in good health, it is relevant to identify early risk factors for premature employment exit throughout the working career.

Cardiovascular disease (CVD) and diabetes are known risk factors for premature employment exit through unemployment, work disability, and early retirement (9). For instance, in a Finnish cohort study, CVD and diabetes were associated with a 3- and 2-fold increased risk for work disability during seven years of follow-up, respectively (10). It is unclear whether an early-stage modifiable cardio-metabolic risk factor like the metabolic syndrome (MetS) already increases the risk for premature employment exit. For a MetS classification, at least three out of the following five risk factor components need to be present: abdominal obesity, hypertension, raised triglycerides, reduced high-density lipoprotein (HDL) cholesterol, and raised blood glucose (11). MetS has been declared an emerging global epidemic that is associated with the worldwide increase of obesity and diabetes (12). MetS prevalence rates in Europe and the United States are estimated to range from 13.2% up to 39.0% depending on the study population and the applied MetS definition criteria (13). The development of MetS differs by socioeconomic factors and is closely linked to modifiable health behaviors, such as smoking, physical inactivity, alcohol consumption, and an unhealthy diet (14, 15). Further, a MetS classification is associated with a 5-fold increased risk for developing type two diabetes mellitus (T2DM) and a 2-fold increased risk for developing CVD over the next 5–10 years (11).

A recent study among Swedish workers aged 15–64 years found that workers with MetS are at 1.4-fold increased risk for work disability after adjustment for sociodemographic, work, and health-related factors (16). MetS might lead to premature employment exit due to its adverse health impact. For instance, hypertension and obesity have been linked to health complaints such as headaches and chronic pain, respectively (17, 18). Further, Burton et al (19) found that classifying for an increasing number of MetS components is associated with an increasing prevalence of sickness absence days. Compared to workers without Mets who reported ≥3 absence days in the previous year (26.5%), the reported percentage of ≥3 absence days among workers who classified for three, four or all five MetS components was 34.2%, 35.7%, and 39.9%, respectively (19). Lastly, a literature review showed the negative impact of MetS on multiple cognitive domains such as memory, processing, or overall intellectual functioning (20). Consequently, MetS might increase the risk for premature employment exit due to its detrimental impact on cognitive aspects, which are crucial for work ability and productivity.

To the best of our knowledge, MetS – as an early-stage (ie, pre-disease) cardio-metabolic health measure that might increase the risk for unemployment or early retirement – has not been examined yet. Moreover, little is known about a possible dose–response relationship between an increasing number of MetS components and the risk for premature employment exit. If MetS and an increasing number of its components are identified as early-stage risk factors for premature employment exit, this knowledge would be of relevance for workers with MetS, employers, and occupational health professionals and may add important insight into which modifiable factors can be addressed to prolong working lives.

The aims of this study are to examine whether (i) MetS increases the risk for premature employment exit through unemployment, work disability, and early retirement and (ii) a dose–response relationship exists between an increasing number of MetS components and the risk for premature employment exit among middle-aged and older workers.

## Methods

### Study design and sample

The present longitudinal study was embedded within the large-scale Lifelines Cohort Study and Biobank (21, 22). Lifelines is a multi-disciplinary prospective population-based cohort study examining the health and health behaviors of 167 729 persons living in the north of The Netherlands (baseline age: 0–93 years). The Lifelines population is broadly representative of the population of the north of The Netherlands on sociodemographic characteristics, lifestyle factors, chronic disease prevalence, and general health (23). Lifelines employs a broad range of investigative procedures in assessing the biomedical, socio-demographic, behavioral, physical, and psychological factors, which contribute to the health and disease of the general population. Eligible participants and their family members were recruited through general practitioners and online self-registration. The ongoing data collection started in 2007 with a comprehensive baseline assessment (T0) at a Lifelines research center, including filling out extensive questionnaires, collecting biological samples, and a physical examination (21, 22). On average, every 1.5 years, follow-up questionnaires are completed (T1, T2, and T4). Approximately five years after T0, participants re-visited a Lifelines research center for the second comprehensive assessment (T3) (21, 22). Approximately 71% of participants took part in both comprehensive assessments, T0 and T3. The response rates for the follow-up questionnaires T1, T2, and T4 were 84%, 68%, and 58%, respectively (22). The current study includes information from the first five measurement waves (T0-T4) with a total median follow-up time of 4.2 [standard deviation (SD) 1.9] years.

The inclusion criteria for the present study were as follows: middle-aged and older workers at T0 (ie, 40–64 years old and working ≥12 hours per week following the Dutch definition of employment at the time of the data collection) (24) who have information on ≥1 follow-up employment status measure after T0. At T0, the Lifelines adult study population (aged 18–93 years) consisted of N=152 728 participants. From this study population, we excluded a total of N=92 301 participants because they did not meet the age criterion (42.6%, N=65 106), worked <12 hours per week at T0 (13.2%, N=20 159), were lost to follow-up after T0 (3.4%, N=5258), or did not have any follow-up measure of employment status after T0 (0.4%, N=679). Additionally, participants in the category educational level ‘other’ (0.7%, N=1023) were excluded as this category is potentially more diverse than the other categories. Lastly, we excluded participants in the International Standard Classification of Occupations (ISCO) 08 category ‘armed forces’ (0.05%, N=76) as there is no task specified for this category (25, 26). The final study sample includes N=60 427 participants.

### Measures

*Premature employment exit.* Employment status was self-reported at each assessment wave by the question ‘Which situation applies to you?’ including the following answer options: paid work (including the number of hours per week), unemployment (registered with the job center), work disability, homemaker, student, retirement, early retirement, or other. Participants could choose several answer options and paid work was given priority in the coding over the other response options. Based on the first self-reported employment status change after T0, the following premature employment exit groups were defined: unemployment (ie, being eligible for work but out of employment while actively searching for work), work disability (ie, having a long-lasting illness leading to an inability to execute or obtain employment), and early retirement (ie, retiring before reaching the state pension age) (27–29). Participants who self-reported to be continuously in employment during the four follow-up waves (T1-T4) built the working control group. Participants who reached the state pension age of 65 years at the time of the data collection or became economically inactive (ie, reported employment status homemaker, student, or other) were censored. Participants with missing values on employment status during follow-up were included if at least one follow-up employment status after T0 is known.

*Metabolic syndrome.* MetS was assessed at T0 using the joint interim criteria (11). For a MetS classification, ≥3 out of the following components need to be present: abdominal obesity [waist circumference (WC) ≥88 cm in females, WC ≥102 cm in males], hypertension (systolic ≥130 and/or diastolic ≥85 mm Hg) or hypertension treatment [anatomical therapeutic chemical (ATC) code C02, C03, C07, C08, or C09], raised triglycerides (≥1.7 mmol/L) or medication to treat lipid abnormalities (ATC code C10A or C10B), reduced HDL-cholesterol (<1.0 mmol/L in males, <1.3 mmol/L in females) or medication to treat lipid abnormalities (ATC code C10A or C10B), raised blood glucose (fasting ≥5.6 mmol/L, non-fasting ≥11 mmol/L) or medication for T2DM (ATC codes A10A or A10B) (11, 30). MetS components were measured by trained research staff using standardized protocols and calibrated measuring instruments (21). WC was measured in the middle between the front end of the lower ribs and the iliac crest in an upright position. Triglycerides, HDL-cholesterol, and glucose were assessed using blood samples. Systolic and diastolic blood pressure was based on the mean value of the final three out of ten measurements from an automatic blood pressure monitor (31).

*Sociodemographic factors*. Age, sex, partner status, educational level, weekly working hours, and occupational group were self-reported at T0. *Partner status* was categorized as married/partnered and not married/partnered. *Educational level* was categorized as low (no education; primary education; lower or preparatory secondary vocational education; junior general secondary education), medium (secondary vocational education or work-based learning; senior general secondary education, pre-university secondary education), or high (higher vocational education; university education) (32). *Weekly working hours* were measured on a scale of 12– 40 hours per week. *Occupational group* was coded according to the ISCO-08 by Statistics Netherlands (25, 33). Based on the major ISCO-08 categories, four overarching occupational groups were compiled: high skilled white-collar (ie, managers, professionals, technicians and associate professionals), low skilled white-collar (ie, clerical support workers, services and sales workers), high skilled blue-collar (ie, skilled agricultural forestry and fishery workers, craft and related trades workers), and low skilled blue-collar workers (ie, plant and machine operators and assemblers, elementary occupations) (34).

*Multiple imputation.* Multiple imputation was carried out to handle missing data on occupational group (N=1617), weekly working hours (N=577), MetS components (highest missing N=503), educational level (N=44), and partner status (N=26). Imputations were predicted by follow-up time, age, gender, and follow-up employment status. In total, ten datasets were imputed (35).

### Statistical analyses

First, baseline MetS, (number of) MetS components, and sociodemographic characteristics were examined by premature employment exit type and for the working control group. Second, the effect of MetS on premature employment exit was investigated using Fine & Gray’s proportional sub distribution hazards survival analysis (36). This analysis is suitable to examine competing exit routes from employment (32, 37). By controlling for competing employment exit types, the analysis acknowledges that one employment exit type ‘prevents’ the occurrence of other employment exit types. Sub distribution hazard ratios (SHR) and corresponding 95% confidence intervals (CI) were presented for MetS and sociodemographic factors. An individual time-to-event variable, reflecting the follow-up time in months until premature employment exit, censoring, or staying in employment, is by default included in the analysis to take the different follow-up times of participants into account. In model 1, the crude association between MetS and premature employment exit types was examined. In model 2, the association between MetS and premature employment exit types was adjusted for sociodemographic factors. This analysis was repeated for the number of classified MetS components (ranging 0–5) to examine whether a dose–response relationship exists between an increasing number of MetS components and premature employment exit types. The MetS classification cut-off value of three MetS components was used as the reference category. Data preparation, multiple imputation of missing values, and descriptive analyses were performed using SPSS Statistics version 28 (IBM Corp, Armonk, NY, USA). Competing risk regression analyses were performed using StataMP 13.1 (StataCorp, College Station, TX, USA).

### Sensitivity analyses

To examine the robustness of the results, the main analyses were repeated including all working participants (N=64 796). In other words, participants who work ≥1 but <12 hours per week at T0 were included (N=4369), following the international definition of employment (27). Additionally, the individual MetS components were investigated as risk factors for premature employment exit.

## Results

### Sample characteristics

At baseline, the mean age of the study sample was 48.3 (SD 5.8) years, 53.8% of the participants were female (N=32 497), and 17.6% of the participants had MetS (N=10 605) (table 1 and supplementary material www.sjweh.fi/article/4113, table S1). During the median follow-up time of 4.2 (SD 1.9) years, 5.6% (N=3403) participants transitioned from employment to unemployment [mean time to event: 2.9 (SD 1.8) years), 1.6% (N=942) to work disability (mean time to event: 2.9 (SD 1.9) years), and 2.1% (N=1297) to early retirement (mean time to event: 3.1 (SD 1.8) years). A total of 3.9% (N=2354) participants were censored because they reached the age of 65 years and 1.2% (N=752) participants were censored because they became economically inactive (N=524 homemakers, N=36 students, and N=192 employment status ‘other’). Participants who transitioned from employment to early retirement during follow-up, were on average ten years older at baseline [59.2 (SD 2.7) years] than participants who transitioned to unemployment [48.9 (SD 5.8) years] or work disability [49.8 (SD 5.9) years]. Compared to the study sample, participants who were lost to follow-up (N=5258) or did not have any follow-up measure of employment status after T0 (N=679) had a slightly higher MetS prevalence at baseline, and were more likely to be lower educated and blue-collar workers (supplementary table S1). Compared to the total T0 adult Lifelines population (22), participants in this study sample are slightly older, and somewhat more likely to be male and lower educated.

**Table 1:** Baseline (T0) characteristics of the working control group and by premature employment exit type during 4.2 years follow-up. [SD=standard deviation; MetS=metabolic syndrome; HDL=high-density lipoprotein]

Baseline			Working control group (N=51 679)			Premature employment exit type during 4.2 years follow-up							
						Unemployment (N=3403)			Work disability (N=942)			Early retirement (N=1297)	
			%	Mean (SD)		%	Mean (SD)		%	Mean (SD)		%	Mean (SD)
Health status													
	MetS		16.5			20.6			29.5			27.6	
	MetS components												
		Abdominal obesity	35.4			40.4			49.9			40.1	
		Hypertension	43.5			47.4			54.4			59.1	
		Raised triglycerides	20.3			22.5			30.7			28.9	
		Reduced HDL-Cholesterol	16.0			19.3			28.0			22.7	
		Raised blood glucose	12.9			16.6			20.3			23.3	
	Number of MetS components												
		0	32.7			28.0			20.3			21.7	
		1	31.3			30.2			27.5			28.1	
		2	19.5			21.2			22.7			22.6	
		3	9.9			11.4			13.1			14.3	
		4	4.8			6.6			11.1			8.6	
		5	1.8			2.6			5.3			4.8	
Sociodemographic factors													
	Age (years)			47.4 (4.9)			48.9 (5.8)			49.8 (5.9)			59.2 (2.7)
	Sex												
		Female	53.7			57.2			56.8			47.6	
		Male	46.3			42.8			43.2			52.4	
	Partner status												
		Married / partnered	90.4			84.5			84.6			93.1	
		Not married / partnered	9.6			15.5			15.4			6.9	
	Occupational group												
		High skilled white-collar	50.6			41.4			34.8			60.1	
		Low skilled white-collar	30.4			38.7			33.2			27.0	
		High skilled blue-collar	10.8			9.9			16.1			7.6	
		Low skilled blue-collar	8.1			9.9			15.8			5.2	
	Educational level												
		High	31.8			23.6			18.7			38.2	
		Medium	41.7			39.1			35.9			27.1	
		Low	26.5			37.3			45.4			34.7	
		Weekly working hours		31.7 (8.6)			30.7 (8.8)			28.2 (9.7)			29.6 (8.8)

### Baseline MetS status and the risk of premature employment exit during follow-up

Baseline MetS prevalence differed by 4.2-year follow-up employment states (table 1). The highest baseline MetS prevalence was present among participants who became work disabled (29.5%), followed by early retirement (27.6%), unemployment (20.6%), and lastly the working control group (16.5%).

In the crude analysis, MetS increased the risk for all premature employment exits types: work disability (SHR 2.01, 95% CI 1.74–2.31), unemployment (SHR 1.24, 95% CI 1.14–1.34), and early retirement (SHR 1.82, 95% CI 1.61–2.06) (table 2, model 1). After adjusting for sociodemographic factors, the associations between MetS and work disability (adjusted SHR 1.78, 95% CI 1.54–2.05) and unemployment (adjusted SHR 1.16, 95% CI 1.06–1.26) were attenuated but remained statistically significant, while the associations for early retirement were not significant anymore (table 2, Model 2).

**Table 2:** The association between metabolic syndrome (MetS) and premature employment exit: competing risk regression analysis. [SHR=sub distribution hazard ratio; CI=confidence interval; HSWC=high skilled white-collar; LSWC=low skilled white-collar; HSBC=high skilled blue-collar; LSBC=low skilled blue-collar; Ref=reference group]. **BOLD indicates statistically significant (P<0.05)**.

Baseline		Premature employment exit type during 4.2 years follow-up										
		Unemployment				Work disability				Early retirement		
		Model 1 ^a^		Model 2 ^b^		Model 1 ^a^		Model 2 ^b^		Model 1 ^a^		Model 2 ^b^
		SHR (95% CI)		SHR (95% CI)		SHR (95% CI)		SHR (95% CI)		SHR (95% CI)		SHR (95% CI)
MetS		**1.24 (1.14–1.34)**		**1.16 (1.06–1.26)**		**2.01 (1.74–2.31)**		**1.78 (1.54–2.05)**		**1.82 (1.61–2.06)**		1.11 (0.97–1.26)
Age (years)				**1.01 (1.00–1.01)**				**1.02 (1.01–1.03)**				**1.37 (1.36–1.38)**
Male sex				0.94 (0.86–1.04)				**1.32 (1.07–1.62)**				0.99 (0.86–1.14)
Not married / partnered				**1.59 (1.44–1.75)**				**1.70 (1.42–2.03)**				**0.54 (0.43–0.68)**
Occupation												
	HSWC			Ref.								
	LSWC			**1.30 (1.19–1.42)**				1.07 (0.89–1.28)				**0.75 (0.64–0.87)**
	HSBC			0.95 (0.83–1.09)				**1.94 (1.56–2.42)**				**0.61 (0.48–0.76)**
	LSBC			1.13 (0.99–1.30)				**1.67 (1.33–2.10)**				**0.47 (0.35–0.62)**
Education												
	High			Ref.								
	Medium			**1.21 (1.10–1.34)**				1.19 (0.97–1.45)				**0.83 (0.72–0.97)**
	Low			**1.60 (1.43–1.79)**				**1.69 (1.36–2.10)**				0.87 (0.74–1.02)
Working hours				1.00 (0.99–1.00)				**0.95 (0.94–0.96)**				**0.99 (0.98–0.99)**

^a^ Model 1 = crude. ^b^ Model 2 = model 1 adjusted for age, sex, occupational group, education, and working hours.

### Increasing number of baseline MetS components and the risk of premature employment exit during follow-up

The number of baseline MetS components classifications differed by 4.2-year follow-up employment states. Classifying for the MetS cut-off value, ie, three components, was most common among participants who became early retirees during follow-up (14.3%). Classifying for four (11.1%) and five MetS components (5.3%) was most common among participants who became work disabled during follow-up. Regarding the specific MetS components, the lowest baseline prevalence rates were present among the working control group, ranging from 12.9% for raised blood glucose to 43.5% for hypertension (table 1). Hypertension and abdominal obesity were most prevalent among workers who prematurely exited employment during follow-up through early retirement (59.1%) and work disability (49.9%), respectively.

A dose–response relationship was found for an increasing number of MetS components and the risk for work disability (table 3). After adjustment for sociodemographic factors, the relationships remained statistically significant and the highest risk for work disability was present among participants who classified for all five MetS components (adjusted SHR 1.81, 95% CI 1.29–2.52). No associations were found between an increasing number of MetS components and unemployment or early retirement after adjusting for sociodemographic factors.

**Table 3:** The association between number of metabolic syndrome (Met)S components and premature employment exit: competing risk regression analysis. [SHR=sub distribution hazard ratio; CI=confidence interval; HSWC=high skilled white-collar; LSWC=low skilled white-collar; HSBC=high skilled blue-collar; LSBC=low skilled blue-collar; Ref=reference group]. **BOLD indicates statistically significant (P<0.05).**

Baseline		Premature employment exit type during 4.2 years follow-up										
		Unemployment				Work disability				Early retirement		
		Model 1 ^a^		Model 2 ^b^		Model 1 ^a^		Model 2 ^b^		Model 1 ^a^		Model 2 ^b^
		SHR (95% CI)		SHR (95% CI)		SHR (95% CI)		SHR (95% CI)		SHR (95% CI)		SHR (95% CI)
MetS components												
	0	**0.78 (0.69–0.88)**		**0.84 (0.74–0.95)**		**0.49 (0.39–0.62)**		**0.55 (0.44–0.70)**		**0.48 (0.40–0.58)**		0.90 (0.74–1.10)
	1	**0.87 (0.78–0.98)**		0.91 (0.81–1.02)		**0.69 (0.56–0.86)**		**0.74 (0.60–0.91)**		**0.65 (0.54–0.77)**		0.89 (0.74–1.07)
	2	0.96 (0.85–1.09)		0.97 (0.85–1.09)		0.89 (0.71–1.11)		0.91 (0.73–1.13)		**0.82 (0.68–0.98)**		0.97 (0.80–1.18)
	3	Ref.										
	4	1.15 (0.98–1.36)		1.13 (0.96–1.33)		**1.70 (1.31–2.20)**		**1.62 (1.25–2.10)**		1.19 (0.94–1.50)		1.01 (0.79–1.30)
	5	1.14 (0.90–1.43)		1.05 (0.83–1.33)		**2.06 (1.48–2.86)**		**1.81 (1.29–2.52)**		**1.71 (1.28–2.28)**		1.06 (0.78–1.44)
Age (years)				**1.01 (1.00–1.01)**				**1.02 (1.01–1.03)**				**1.37 (1.36–1.38)**
Male sex				0.94 (0.85–1.03)				**1.29 (1.05–1.58)**				0.99 (0.86–1.14)
Not married / partnered				**1.59 (1.44–1.74)**				**1.67 (1.40–2.00)**				**0.54 (0.43–0.68)**
Occupation												
	HSWC			Ref.								
	LSWC			**1.30 (1.19–1.42)**				1.07 (0.89–1.28)				**0.75 (0.64–0.87)**
	HSBC			0.95 (0.83–1.09)				**1.96 (1.57–2.44)**				**0.61 (0.48–0.77)**
	LSBC			1.13 (0.98–1.30)				**1.64 (1.31–2.06)**				**0.47 (0.35–0.62)**
Education												
	High			Ref.								
	Medium			**1.20 (1.09–1.33)**				1.15 (0.94–1.41)				**0.83 (0.71–0.97)**
	Low			**1.58 (1.42–1.76)**				**1.61 (1.30–2.00)**				0.87 (0.74–1.02)
Working hours				1.00 (0.99–1.00)				**0.95 (0.94–0.96)**				**0.99 (0.98–0.99)**

^a^ Model 1 = crude. ^b^ Model 2 = model 1 adjusted for age, sex, occupational group, education, and working hours.

### Sensitivity analyses

The main study findings were similar when all working participants, ie, also participants who worked ≥1 but <12 hours per week at baseline (N=4369), were included in the analysis (supplementary tables S2 and S3). Further, all MetS components except raised blood glucose were identified as risk factors for work disability, while only raised blood glucose was identified as a risk factor for unemployment (supplementary table S4, Model 2).

## Discussion

In this 4.2 year follow-up study among 60 427 middle-aged and older Dutch workers, MetS increased the risk for premature employment exit especially through work disability and to a lesser extent through unemployment. Specifically, middle-aged and older workers with MetS at baseline were 1.8-times more likely to become work disabled during follow-up. Further, a clear dose–response relationship was found for an increasing number of MetS components and the risk for work disability. In other words, workers who classified for four or all five MetS components had a higher risk to become work disabled during follow-up than workers who classified for three MetS components. Further, workers with MetS at baseline were 1.2-times more likely to become unemployed during follow-up. No dose–response relationship was found between an increasing number of MetS components and unemployment. Neither MetS nor an increasing number of MetS components was associated with early retirement in this study. The total study sample MetS prevalence was 17.6% which is comparable to MetS prevalence rates found in other European countries (13).

The finding that MetS increased the risk for work disability corresponds with the result of a recent population-based prospective cohort study among Swedish employees (16). The present study is the first to show that MetS also increases the risk for unemployment. Taken together, these findings suggest that not only chronic diseases like CVD and T2DM are risk factors for premature employment exit (9), but that MetS as a modifiable early-stage cardio-metabolic risk factor is already an important indicator for future work participation. It is worth mentioning that the overall unemployment rate was slightly higher in the catchment area of the current study (ie, Groningen, Friesland, and Drenthe) during the time of data collection compared to the rest of The Netherlands (38). Consequently, the chance to become unemployed might have been somewhat higher for the study participants compared to workers in other regions of The Netherlands. The finding that neither MetS nor an increasing number of MetS components was associated with early retirement suggests that early retirement seems to be less dependent on cardio-metabolic risk factors than work disability or unemployment. For early retirement, other factors such as the voluntariness of premature employment exit or age might play a more important role. First, early retirement can generally be considered a more voluntary choice to stop working, compared to work disability and unemployment where workers might be involuntarily forced to exit employment due to ill-health (5). Second, participants who transitioned into early retirement were on average ten years older at baseline compared to the total study sample and MetS prevalence increases with age (39).

The current study findings may inform policy and practice. More awareness is needed among employers and occupational health professionals about the premature employment exit risk faced by middle-aged and older workers with MetS. Further, MetS and MetS components might be included as modifiable risk factors in interventions to prevent work disability and unemployment. As this is one of the first studies investigating MetS as an early-stage risk factor for premature employment exit types, more research is needed. It might be of particular interest to gain more insight into which MetS component clusters act as main drivers for premature employment exit and to elaborate on the specific pathways and mechanisms between MetS and premature employment exit through work disability and unemployment. Possible linking mechanisms might be the adverse effect of MetS on work productivity, cognitive functioning, and increased absence days (19, 40).

Study strengths are the longitudinal design with a clear temporal order of measurements, which minimizes the risk of reverse causation. Further, MetS components were measured by trained research staff and included information on medication use, which reduces the risk of information bias. Lastly, although more comparative research is needed from other geographical areas, the Lifelines population is broadly representative of the population of the north of The Netherlands, which facilitates the generalizability of results (23). A study limitation concerns the possibility of misclassification of premature employment exit types as employment states were self-reported. However, previous research suggests high agreement between self-reported and register-based employment history data (41). Another limitation is the limited follow-up time of 4.2 years and that employment states have only been assessed every 1.5 years. Consequently, it cannot be ruled out that participants experienced transitions between premature employment exit types or temporarily left employment between two assessment points. Further, participants may leave or re-enter employment, which is more likely to be captured with a longer follow-up period. Moreover, the limited follow-up time in combination with the baseline mean age of 48.3 years makes the premature employment exit types unemployment and work disability more likely to occur than early retirement in the current study. The use of longer follow-up data and linkages with register data on employment status in future studies would allow to investigate long-term effects of MetS on work participation and more detailed employment trajectories. Lastly, a slight underestimation of MetS prevalence and some selection bias may have occurred as participants who were lost to follow-up or did not have any follow-up measure of employment status were slightly unhealthier and more likely to be lower educated than study sample participants.

In conclusion, MetS was identified as a modifiable early-stage cardio-metabolic risk factor for premature employment exit especially through work disability and to a lesser extent through unemployment. Further, a clear dose–response relationship was found for an increasing number of MetS components and the risk for work disability. MetS prevention and MetS interventions might help to prolong working lives. More awareness is needed among employers and occupational health professionals about the premature employment exit risk faced by middle-aged and older workers with MetS.

## Supplementary material

Supplementary material

## Data Availability

The Lifelines Cohort Study is conducted according to the principles of the Declaration of Helsinki and in accordance with the research code of the University Medical Center Groningen (UMCG). The medical ethical committee of UMCG, The Netherlands approved the Lifelines study (ethics number: 2007/152). Participants gave informed consent to participate in the study before taking part. Data are available on reasonable request. Data may be obtained from a third party and are not publicly available. Researchers can apply for the data and biomaterial through a proposal that is submitted to the Lifelines research office (research@lifelines.nl).
