# Supplementary material for: Metabolic syndrome increases the risk for premature employment exit: A longitudinal study among 60 427 middle-aged and older workers from the Lifelines Cohort Study and Biobank
Source: Scand J Work Environ Health. 2023 Oct 31;49(8):569–77. doi: 10.5271/sjweh.4113 (PMC10866619; doi:10.5271/sjweh.4113)
Supplement: Supplementary material [file SJWEH-49-569-S001.pdf]

Metabolic syndrome increases the risk for premature employment exit: A longitudinal study among 60 427 middle-aged and older workers from the Lifelines Cohort Study and Biobank[1]

by Katharina Runge, MSc,[2] Sander KR van Zon, PhD, Kène Henkens, PhD, Ute Bültmann, PhD

1. *Supplementary tables*
2. *Correspondence to: Katharina Runge, Department of Health Sciences, Community and Occupational Medicine, University of Groningen, University Medical Center Groningen, Hanzeplein 1, 9700 RB Groningen, The Netherlands. [E-mail: k.runge@umcg.nl]*

Table S1: Comparison of the study sample baseline (T0) characteristics with participants who dropped-out after T0 or had no follow-up information on employment status (ES)

	**Study sample** N=60 427		**Drop-out sample** N=5258		**Missing ES sample** N=679	
Baseline	%	mean (SD)	%	mean (SD)	%	mean (SD)
**Health status**						
MetS	17.6		18.1		20.3	
MetS components						
Abdominal obesity	36.3		40.5		36.8	
Hypertension	44.9		47.1		43.0	
Raised triglycerides	21.0		20.2		24.6	
Reduced HDL-Cholesterol	16.7		16.6		18.4	
Raised blood glucose	13.9		13.0		15.2	
Number of MetS components						
0	31.5		24.4		30.2	
1	31.0		27.1		29.6	
2	19.9		18.7		17.1	
3	10.3		10.3		11.9	
4	5.2		5.7		5.7	
5	2.1		2.0		2.7	
**Sociodemographic factors**						
Age (years)		48.3 (5.8)		46.5 (5.0)		46.8 (5.0)
Sex						
Female	53.8		49.5		43.6	
Male	46.2		50.5		56.4	
Partner status						
Married / partnered	90.0		87.8		88.4	
Not married / partnered	10.0		12.1		11.5	
Occupational group						
High skilled white-collar	49.8		41.2		45.1	
Low skilled white-collar	31.1		30.8		25.0	
High skilled blue-collar	10.8		12.6		12.4	
Low skilled blue-collar	8.3		11.4		12.5	
Educational level						
High	31.1		24.3		27.2	
Medium	40.5		38.2		37.4	
Low	28.4		35.9		31.7	
Weekly working hours		31.4 (8.7)		32.4 (8.7)		33.0 (8.5)

Abbreviations: SD, standard deviation; MetS, metabolic syndrome; HDL, high-density lipoprotein

Table S2: The association between MetS and premature employment exit: competing risk regression analysis (sensitivity analysis: including participants working ≥1 hour but <12 hours per week, N=64 796)

	**Premature employment exit type during 4.2 years follow-up**					
	**Unemployment**		**Work disability**		**Early retirement**	
Baseline	Model 1 SHR (95% CI)	Model 2 SHR (95% CI)	Model 1 SHR (95% CI)	Model 2 SHR (95% CI)	Model 1 SHR (95% CI)	Model 2 SHR (95% CI)
MetS	**1.21 (1.12-1.31)**	**1.14 (1.05-1.23)**	**2.05 (1.81-2.33)**	**1.80 (1.58-2.04)**	**1.73 (1.54-1.94)**	1.04 (0.92-1.18)
**Covariates**						
Age (years)		1.00 (1.00-1.01)		**1.01 (1.00-1.02)**		**1.36 (1.35-1.37)**
Male sex		0.98 (0.90-1.07)		**1.38 (1.16-1.63)**		0.96 (0.84-1.09)
Not married / partnered		**1.64 (1.50-1.79)**		**1.88 (1.60-2.20)**		**0.54 (0.43-0.68)**
Occupation						
HSWC		Ref.				
LSWC		**1.32 (1.22-1.44)**		1.04 (0.88-1.22)		**0.73 (0.63-0.84)**
HSBC		0.96 (0.84-1.10)		**1.76 (1.43-2.17)**		**0.59 (0.48-0.74)**
LSBC		**1.22 (1.08-1.38)**		**1.37 (1.12-1.69)**		**0.49 (0.38-0.62)**
Education						
High		Ref.				
Medium		**1.17 (1.07-1.29)**		**1.25 (1.04-1.50)**		0.87 (0.76-1.01)
Low		**1.54 (1.39-1.71)**		**1.72 (1.41-2.10)**		0.87 (0.75-1.02)
Working hours		**1.00 (0.99-1.00)**		**0.95 (0.95-0.96)**		1.00 (0.99-1.01)

Abbreviations: SHR, sub distribution hazard ratio; CI, confidence interval; MetS, metabolic syndrome; HSWC, high skilled white-collar; LSWC, low skilled white-collar; HSBC, high skilled blue-collar; LSBC, low skilled blue-collar; Ref., reference group

Note: model 1 = crude; model 2 = model 1 adjusted for age, sex, occupational group, education, and working hours; SHR's written in bold are statistically significant (p<.05)

Table S3: The association between number of MetS components and premature employment exit: competing risk regression analysis (sensitivity analysis: including participants working ≥1 hour but <12 hours per week, N=64 796)

	**Premature employment exit type during 4.2 years follow-up**					
	**Unemployment**		**Work disability**		**Early retirement**	
Baseline	Model 1 SHR (95% CI)	Model 2 SHR (95% CI)	Model 1 SHR (95% CI)	Model 2 SHR (95% CI)	Model 1 SHR (95% CI)	Model 2 SHR (95% CI)
MetS components						
0	**0.80 (0.72-0.90)**	**0.86 (0.77-0.96)**	**0.46 (0.38-0.56)**	**0.53 (0.43-0.64)**	**0.51 (0.43-0.61)**	0.98 (0.82-1.19)
1	0.90 (0.81-1.01)	0.94 (0.84-1.05)	**0.63 (0.52-0.76)**	**0.68 (0.57-0.83)**	**0.69 (0.58-0.82)**	0.96 (0.81-1.15)
2	0.98 (0.87-1.11)	0.99 (0.88-1.11)	**0.79 (0.65-0.96)**	**0.81 (0.67-0.99)**	0.86 (0.72-1.03)	1.05 (0.87-1.26)
3	Ref.					
4	**1.17 (1.00-1.37)**	1.15 (0.98-1.35)	**1.46 (1.16-1.85)**	**1.43 (1.13-1.80)**	1.21 (0.96-1.51)	1.09 (0.86-1.38)
5	1.13 (0.91-1.41)	1.05 (0.84-1.31)	**1.81 (1.35-2.43)**	**1.55 (1.15-2.09)**	**1.71 (1.30-2.25)**	1.05 (0.79-1.41)
**Covariates**						
Age (years)		1.00 (1.00-1.01)		1.01 (1.00-1.02)		**1.36 (1.35-1.37)**
Male sex		0.97 (0.89-1.06)		**1.34 (1.14-1.59)**		0.96 (0.84-1.09)
Not married / partnered		**1.64 (1.50-1.79)**		**1.86 (1.58-2.18)**		**0.54 (0.43-0.68)**
Occupation						
HSWC		Ref.				
LSWC		**1.32 (1.21-1.44)**		1.03 (0.87-1.22)		**0.73 (0.63-0.84)**
HSBC		0.96 (0.84-1.10)		**1.77 (1.43-2.18)**		**0.60 (0.48-0.74)**
LSBC		**1.21 (1.07-1.37)**		**1.35 (1.09-1.66)**		**0.49 (0.38-0.62)**
Education						
High		Ref.				
Medium		**1.16 (1.06-1.28)**		**1.21 (1.01-1.46)**		0.87 (0.75-1.01)
Low		**1.52 (1.37-1.69)**		**1.65 (1.35-2.02)**		0.87 (0.75-1.01)
Working hours		**1.00 (0.99-1.00)**		**0.95 (0.95-0.96)**		1.00 (0.99-1.01)

Abbreviations: SHR, sub distribution hazard ratio; CI, confidence interval; MetS, metabolic syndrome; HSWC, high skilled white-collar; LSWC, low skilled white-collar; HSBC, high skilled blue-collar; LSBC, low skilled blue-collar; Ref., reference group

Note: model 1 = crude; model 2 = model 1 adjusted for age, sex, occupational group, education, and working hours; SHR's written in bold are statistically significant (p<.05)

Table S4: The association between MetS components and premature employment exit: competing risk regression analysis

	Premature employment exit type					
	Unemployment		Work disability		Early retirement	
	Model 1<br>SHR (95% CI)	Model 2<br>SHR (95% CI)	Model 1<br>SHR (95% CI)	Model 2<br>SHR (95% CI)	Model 1<br>SHR (95% CI)	Model 2<br>SHR (95% CI)
**MetS components**						
Abdominal obesity	**1.17 (1.09-1.26)**	1.08 (1.00-1.16)	**1.53 (1.34-1.75)**	**1.37 (1.19-1.58)**	0.99 (0.88-1.11)	0.99 (0.88-1.12)
Hypertension	1.05 (0.98-1.13)	1.03 (0.96-1.11)	**1.22 (1.07-1.40)**	**1.15 (1.00-1.33)**	**1.60 (1.43-1.80)**	1.05 (0.93-1.18)
Raised triglycerides	0.98 (0.89-1.08)	1.00 (0.91-1.10)	**1.18 (1.01-1.38)**	**1.20 (1.02-1.42)**	**1.19 (1.04-1.35)**	0.98 (0.82-1.16)
Reduced HDL-Cholesterol	**1.12 (1.02-1.24)**	1.08 (0.98-1.20)	**1.50 (1.28-1.76)**	**1.43 (1.21-1.69)**	1.14 (0.99-1.30)	1.17 (0.97-1.40)
Raised blood glucose	**1.16 (1.06-1.28)**	**1.15 (1.04-1.26)**	**1.20 (1.02-1.42)**	1.15 (0.97-1.37)	**1.58 (1.37-1.82)**	1.02 (0.88-1.18)
**Covariates**						
Age (years)		**1.01 (1.00-1.01)**		**1.02 (1.01-1.03)**		**1.37 (1.36-1.38)**
Male sex		0.95 (0.86-1.05)		**1.36 (1.09-1.68)**		0.98 (0.85-1.14)
Not married / partnered		**1.58 (1.44-1.74)**		**1.68 (1.41-2.02)**		**0.54 (0.43-0.68)**
Occupation						
HSWC		Ref.				
LSWC		**1.30 (1.18-1.42)**		1.07 (0.89-1.28)		**0.75 (0.64-0.87)**
HSBC		0.95 (0.83-1.09)		**1.96 (1.58-2.44)**		**0.61 (0.48-0.77)**
LSBC		1.13 (0.98-1.29)		**1.64 (1.30-2.06)**		**0.47 (0.35-0.62)**
Education						
High		Ref.				
Medium		**1.20 (1.09-1.33)**		1.15 (0.94-1.41)		**0.83 (0.71-0.97)**
Low		**1.58 (1.42-1.77)**		**1.61 (1.30-2.00)**		0.87 (0.74-1.02)
Working hours		1.00 (0.99-1.00)		**0.95 (0.94-0.96)**		**0.99 (0.98-0.99)**

Abbreviations: SHR, sub distribution hazard ratio; CI, confidence interval; MetS, metabolic syndrome; HSWC, high skilled white-collar; LSWC, low skilled white-collar; HSBC, high skilled blue-collar; LSBC, low skilled blue-collar; Ref., reference group

Note: model 1 = crude; model 2 = model 1 adjusted for age, sex, occupational group, education, and working hours; SHR's written in bold are statistically significant ($p<.05$)
